# Histopathological Changes and Inflammatory Response in Specific Pathogen-Free (SPF) with Porcine Circovirus Type 3 Infection

**DOI:** 10.3390/ani13030530

**Published:** 2023-02-02

**Authors:** Huidan Deng, Song Zhu, Ling Zhu, Zhijie Jian, Yuancheng Zhou, Fengqin Li, Lishuang Deng, Junliang Deng, Youtian Deng, Siyuan Lai, Zhiwen Xu

**Affiliations:** 1 College of Veterinary Medicine, Sichuan Agricultural University, Chengdu 611130, China; 2 Animal Biology Technology Center, Sichuan Agricultural University, Chengdu 611130, China; 3 Animal Breeding and Genetics Key Laboratory of Sichuan Province, Sichuan Animal Science Academy, Chengdu 610058, China; 4 College of Animal Science, Xichang University, Xichang 615012, China

**Keywords:** porcine circovirus 3 (PCV3), specific pathogen-free (SPF) piglets infection model, pathogenicity

## Abstract

**Simple Summary:**

Since the first report of PCV3 virus infection in 2016, it has been associated with sow mortality, lesions consistent with porcine dermatitis and nephropathy syndrome, reproductive failure, and multisystemic inflammation. In this study, a PCV3 infection model was created using SPF pigs, and the inflammatory response and histological alterations were examined. The results showed that after infection with PCV3, the piglets showed reductions in body weight gain and fever. During the study, viremia, nasal shedding, and fecal shedding were all discovered. Pathological abnormalities were visible in the heart, lung, liver, kidney, lymph nodes, and spleen, including edema, inflammation, cell aging, necrosis, and bleeding. Additionally, the group that received PCV3 inoculation had considerably more pro-cytokines in their serum. The only organs with a high viral load were the heart, lungs, liver, kidney, and lymph nodes.

**Abstract:**

Since the first report of PCV3 virus infection in 2016, it has been linked to multisystemic inflammation, reproductive failure, cardiac pathology, and clinical indications resembling porcine dermatitis and nephropathy syndrome (PDNS). However, the pathogenesis and clinical significance of PCV3 is still unclear. In this study, a PCV3 infection model was created using SPF pigs, and histopathology and fluorescence quantitative PCR were utilized to examine PCV3’s pathogenicity. Reductions in body weight gain and fever were observed during this study. However, other clinical signs such as Dermatitis and Nephropathy Syndrome were not observed through the study. Viremia was detected in the PCV3-inoculated group from 17 days post-inoculation (p.i.) until the end of the study. Nasal shedding was detected from 21 to 35 dpi and fecal shedding was detected during 25–33 days and 39 days, respectively. Gross lesions and histological evaluation were detected in various tissues and organs, including the lung, heart, kidney, lymph nodes, spleen, liver, small intestine, and testis. The heart, lung, liver, kidney, lymph nodes, and spleen showed pathological changes. The pathological features include swelling, inflammation, cell degeneration, necrosis, and hemorrhage. The lesions are consistent with multisystemic inflammation. Tissue viral load results showed only heart, lung, liver, kidney, lymph nodes, and spleen was positive by qRT-PCR. Moreover, the pro-inflammation cytokines in serum increased a lot in the PCV3-inoculated group compared to the control group, demonstrating that the induced inflammation response may be the cause of tissue damage in PCV3-infection. This study demonstrated that PCV3 can produce mild pathological damage to multiple organs, especially multisystemic inflammatory cell infiltration and prolonged viremia, viral shedding in nasal secretions. This is the first in vivo characterization of PCV3 infection in the SPF piglets model using isolated PCV3 strain, and this is also the first time to show the gross and pathological lesion with all tissue and organs in the PCV3-inoculated group. Our findings might serve as a starting point for more investigation into PCV3’s pathogenic mechanism.

## 1. Introduction

Porcine circovirus (PCVs) belongs to the genus Circovirus within the family Circoviridae, and, currently, two porcine circovirus genotypes, PCV1 and PCV2, have been extensively studied. Porcine circovirus type 1 (PCV1) is not pathogenic to pigs and was first discovered to be a contamination of the porcine kidney cell line (PK-15) [1]. Porcine circovirus type 2 (PCV2) is the causative agent of porcine circovirus associated diseases (PCVAD) [2], including respiratory and intestinal diseases, reproductive failure, porcine dermatitis and nephrotic syndrome (PDNS), and PCV2-systemic diseases. PCV2 has caused significant economic loss for the global pig industry [3].

PCV3 was first identified in the United States in 2016 from a farm with elevated sow mortality rate and PDNS-like lesions in sows, chronic reproductive issues manifested as lower conception rates and increased mummification and stillbirth rates [4]. Another report on 3-week-old pigs described the presence of myocarditis from an unidentified source. Viral meta-genomic sequencing revealed the presence of PCV3, and in situ hybridization (ISH) confirmed replication in the myocardiocytes and inflammatory cells associated with myocarditis, as well as arterioles media [5]. Since these initial reports, PCV3 infection and genome presence in pigs have been confirmed in Asia, Europe, and South America [6,7,8,9,10,11]. Although PCV3 can be detected in pigs of different ages, including sows, piglets, adult pigs, mummified fetuses, and stillbirths [4,12], the epidemic survey suggested that the positive rate of PCV3 genome was different among different age groups. The PCV3 infection rate of lactating pigs was lower than that of finishing pigs. The highest PCV3 infection rate was found in weaned piglets [7,13,14].

It has been reported that PCV3 may be associated with a variety of different clinical symptoms or pathological conditions, such as respiratory diseases, reproductive disorders, gastrointestinal diseases, and neurological diseases. However, PCV3 can also be found in healthy animals that are asymptomatic [4,15]. Thus, the pathogenicity and clinical relevance of PCV3 remains unknown. Regarding the studies and reports of PCV3 clinically related diseases, we can find that the majority of these studies lacked comprehensive and extensive diagnostic information and instead just looked at the PCV3 genome in pigs exhibiting various clinical symptoms. This does not directly confirm that PCV3 plays a major role in these clinical symptoms. In order to explore the pathogenicity of PCV3, some scholars have established experimental models using tissue or cytotoxicity of PCV3. Experimental infection of PCV3 cannot reproduce the clinical signs and symptoms as described before. The atomical findings showed tissue structural damage and inflammation. However, the experimental results are conflicting; the pathogenicity of PCV3 is still controversial [16,17,18,19]. Therefore, the establishment of a standard experimental animal model using PCV3 strains is still necessary. It is conducive to a comprehensive and in-depth understanding of the pathogenicity of PCV3.

Considering the uneven quality of model animals used in porcine disease research at home and abroad, there is no uniform standard to use. This may cause potential infection or conditional pathogenic pathogens, interfering with experimental results. Here, specific pathogen-free (SPF) piglets (which have the characteristics of no specific pathogen, stable genetic shape and excellent serum quality) were used to establish the PCV3 infection model. Our results are expected to reveal the pathogenicity of PCV3 and provide a theoretical basis for the clinical diagnosis, prevention, and control of PCV3.

## 2. Materials and Methods

### 2.1. Virus and Animals

Pig (Sus scrofa) kidney epithelial cells (PK-15) were inoculated with 1 mL of tissue homogenate supernatant and incubated for 1.5 h at 37 °C with 5% CO_2_ to allow virus adsorption. Then, 4 mL of medium (EMEM, 1% penicillin/streptomycin (GibcoTM, Thermo Scientific, Waltham, MA, USA)) and 25 µg/mL of gentamicin (GibcoTM, Thermo Scientific) was added, and incubated for an additional 70 h at 37 °C with 5% CO_2_. The virus was harvested in 15 mL conical tubes centrifuged. Last, the supernatant was collected and identified as passage 1. Five serial passages of virus isolated in passage 1 were performed. The final stock of PCV3 (accession number: ON989005) was maintained at Key Laboratory of Animal Disease and Human Health of Sichuan Province, Sichuan Agricultural University, Chengdu.

SPF pigs (28 days old) were purchased from ChongQing Academy of Animal Sciences.

### 2.2. Animal Treatment

Eight 28-day-old specific-pathogen-free (SPF) Rongchang piglets from the ChongQing Academy of Animal Sciences, China, were randomly assigned to two groups (four piglets in the control group and four piglets in the PCV3-inoculated group). Each group of piglets was housed in an individual room and fed sterile food and water ad libitum. Prior to inoculation, all the piglets were shown to be negative for antigens to PCV2, PCV3, pseudorabies virus (PRV), classical swine fever (CSFV), porcine epidemic diarrhea virus (PEDV), Japanese encephalitis virus (JEV), and PRRSV by qPCR. After one week of acclimation, the control group was intramuscular injected with cell culture medium (dose of 3 mL/15 kg body weight), the PCV3-inoculated group was intramuscular injected with 1 × 10^6.5^ genomic copies per μL PCV3 (passage 3). After 42 days of the experiments, all piglets were sacrificed humanely.

### 2.3. Clinical Evaluation

All pigs were weighed every day, and the relative weight gain for each week were determined. Clinical observation including lethargy, respiratory signs, inappetence, icterus, and lameness were recorded daily.

### 2.4. Sample Collection

Blood was collected from all pigs at 0, 3, 7, 10, 14, 17, 21, 23, 25, 27, 29, 31, 33, 35, 37, 39, and 41 days in 5 mL serum separator tubes (ThermoFisher Scientific, Waltham, MA, USA). The blood was centrifuged at 2000 g for 10 min and serum aliquots were stored at −80 °C until testing.

Individual pig nasal and fecal samples were also collected at independent times using polyester swabs (ThermoFisher Scientific, Waltham, MA, USA). The swabs were stored in 5 mL plastic tubes (ThermoFisher Scientific, Waltham, MA, USA), each containing 1 mL of sterile MEM (ThermFisher Scientific, Waltham, MA, USA).

During necropsy at 42 days, a full set of tissues, including the heart, liver, spleen, lung, kidney, tonsil, lymph node, brain, small intestine, testis, and ovary was collected in separate bags at −80 °C and fixed in 4% paraformaldehyde.

### 2.5. Evaluation of Viremia, Viral Shedding and Detection of PCV3 in Tissues by qRT-PCR Assay

PCV3 viral loads in tissues, serum and swabs were measured by quantitative real-time PCR. The total DNA was extracted from different samples using the Cofitt Universal Genomic DNA Kit (LBD9503-50, Hong Kong, China) according to the manufacturer’s instructions. DNA concentration and purity were measured using ScanDrop test A260 value and A260/A280 ratio. Quantitative RT-PCR was carried out on a Roche Lightcycler96 instrument using TB Green Premix Ex Taq according to the Manufacturer’s instructions. The PCV3 primer sequences showed as follows: PCV3-F: TKGATGAYTTTTATGGSTGG; PCV3-R: CTCCTAAACAAGGCCTCCAACT. The PCV3 genome copy number per μL was calculated from a standard curve created by the ten-fold serial dilution of plasmid DNA containing the genome of PCV3. The standard used in this experiment was established in our lab. The standard curve is y = −3.3294x + 37.9. The sensitivity was 5.5 copies/μL, and we use a PCV3 primer to detect PCV2, PRRSV, PRV, CSFV. Only PCV3 can be detected.

### 2.6. Histopathology Evaluation

At the 42 days of experiments, the gross lesion of pathological changes in tissues and organs were observed. Then, the same tissue samples were fixed in 4% paraformaldehyde and subjected to gradient ethanol dehydration and paraffin embedding. Afterwards, the samples were sectioned into slices (thickness, 4-μm) and stained with hematoxylin and eosin (H&E), and histopathological characteristics were observed under an optical microscope.

### 2.7. ELISA of Cytokine

The level of IL-1β, TNF-α, IL-6, IFN-γ, IL-2, and IL-8 in serum at indicated time (0, 7, 14, 21, 35, and 41 days respectively) were detected using Elisa assy. The test was carried out according to the kit brochures operation (Thermo Fisher).

### 2.8. Statistical Analysis

Data were depicted in the manner of mean ± SD. A t-test was employed to explore the significance of difference across three experimental groups and the control group using Graphpad. *p* < 0.05 indicates statistical significance of the difference.

## 3. Results

### 3.1. Clinical Signs Induced by PCV3 Infection

**Figure 1:**
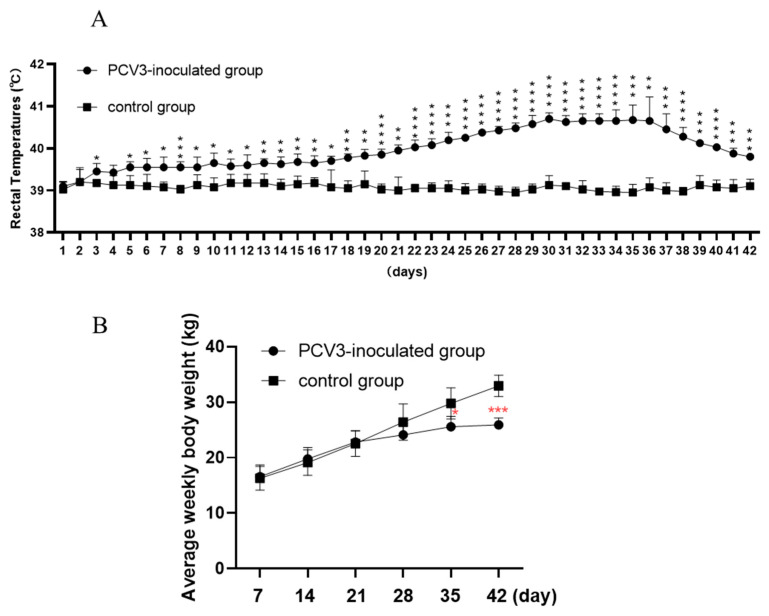
The daily rectal temperature (**A**) and average weekly body weight (**B**) in the control group and PCV3-inoculated group. * means *p* < 0.05, ** means *p* < 0.01, *** means *p* < 0.005, **** means *p* < 0.0001 when compare PCV3-inoculted group with control group.

No obvious clinical signs were observed in the control group. In the PCV3-inoculated group, clinical signs such as loss of appetite, depression, diarrhea, and lameness were first shown in 2 of 4 piglets at the 19 dpi. All the piglets exhibited above mentioned clinical signs at the 23 dpi until the end of the experiment. All the piglets in the PCV3 group exhibited rectal temperatures of 40.0 °C (ranging from 40.0 °C to 41.5 °C) for approximately 18 consecutive days (23–40 dpi) after inoculation (Figure 1A). Following PCV3 inoculation, the piglets in PCV3 groups exhibited reductions in body weight gain after 21 dpi until the end of the experiment (Figure 1B). The temperature changes and body weight gains were roughly consistent with the clinical signs after PCV3 inoculation.

### 3.2. PCV3 Produces Persistent Viremia

Serum samples were serially collected from 0 to 42 days after PCV3 infection and tested for PCV3 detection and quantification by qRT-PCR. All pigs in the control groups tested negative for the presence of PCV3 in serum samples (viremia) throughout the study. In contrast, persistent viremia was detected in all piglets from PCV3-inoculated groups from 23 days until the end of the study. The genome copies was 10^2.05^ copies/μL at 17 dpi and continued to increase. Peak viremia occurred in PCV3 groups at 35 days and maintained constant through the rest of the study. One out of four pigs (25%) in the PCV3-inoculated group had viremia (genome copies was 10^4.46^ copies/μL) at 17 days of the experiments. Two out of four piglets (50%) produce viremia at 21 days, and all pigs in the PCV3- inoculated groups produce viremia after 21 days (Table 1).

### 3.3. PCV3 Produces Shedding in Nasal and Faecal Secretions

Nasal and fecal swabs were also collected from 0 to 42 days. All nasal and fecal swabs collected from both control groups tested negative for PCV3 throughout the study. PCV3 detection in nasal swab samples of PCV3-inoculated pigs showed that the virus can be detected in nasal secretions from 21 to 35 days (Table 2). The highest genomic copies in nasal swab was observed at 31 days. Two out of four pigs (50%) in the PCV3-inoculated group shed the virus in nasal secretions at 21 days, and all pigs in the PCV3-inoculated groups shed the virus in nasal secretions at 29 and 31 days (Table 2). Interestingly, three out of four (75%) pigs in the PCV3-inoculated group showed intermittent patterns of nasal shedding. The average genomic copies of the PCV3-inoculated group ranged from 10^1.76^ to 10^2.99^ copies/μL. The detection of PCV3 in feces was intermittent. The percentage of piglets that tested positive in the inoculated PCV3-im group was 25% (1/4) at 21 and 35 days, 75% (3/4) at 23 days, and 50% (2/4) at 25–33 days and 39 days, respectively. The average genomic copies of feces ranged from 10^1.78^ to 10^2.13^ copies/μL (Table 3). These results indicated that the highest detection of PCV3 in feces occurred during the fourth week after inoculation.

### 3.4. Gross Lesions Caused by PCV3 Infection

**Figure 2:**
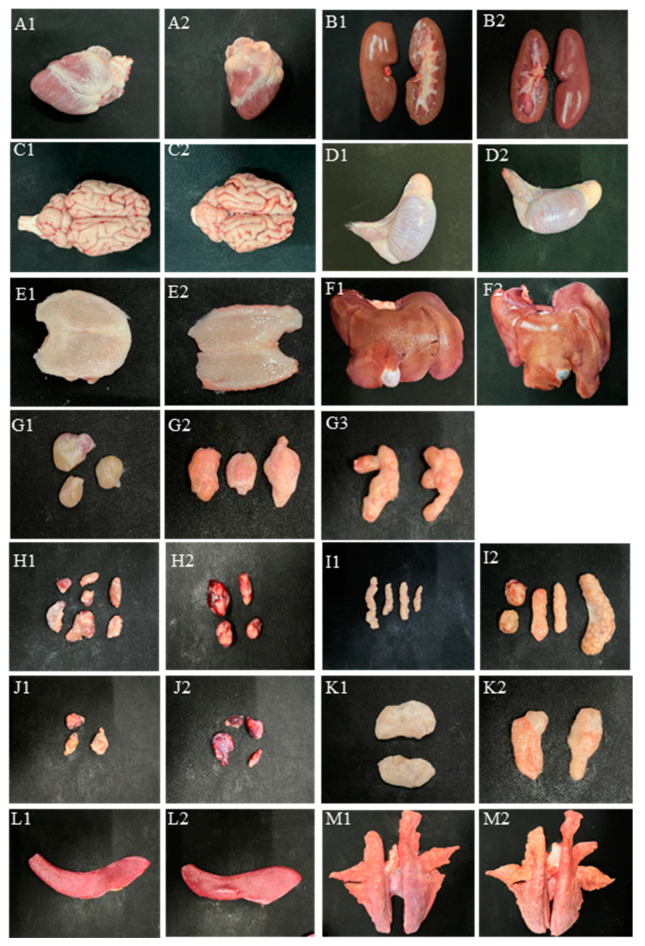
The gross lesions of hearts (**A1**,**A2**), kidneys (**B1**,**B2**), brains (**C1**,**C2**), testis (**D1**,**D2**), tonsil (**E1**,**E2**), livers (**F1**,**F2**), submaxillary lymph node (**G1**–**G3**), hilar lymph node (**H1**,**H2**), mesenteric lymph node (**I1**,**I2**), liver portal lymph node (**J1**,**J2**), inguinal lymph node (**K1**,**K2**), spleen (**L1**,**L2**), and lung (**M1**,**M2**). The number 1 means control group, other numbers represent the PCV3-inoculated group.

From 4-week-old piglets, lungs, livers, kidneys, spleens, hearts, and lymph nodes (inguinal lymph node, submaxillary lymph node, mesenteric lymph node, hilar lymph node and liver portal lymph node), brain, testis, and tonsils were collected from all the groups for gross observations. As shown in Figure 2. When compared with the control group (presented as Figure 2(A1–M1)), the heart (Figure 2(A2)), kidney (Figure 2(B2)), brain (Figure 2(C2)), testis (Figure 2(D2)), and tonsils (Figure 2(E2)) do not exhibit notable macroscopic lesions. Two out of four livers (Figure 2(F2)) were hyperemic, mottled tan-to-purple. Three out of four of the submaxillary lymph node were enlarged, hyperemic (Figure 2(G2)), and showed hyperplasia (Figure 2(G3)), and two out of four of the submaxillary lymph node showed festering focal (Figure 2(G3,G4)). Four out of four of the hilar lymph node (Figure 2(H2)), four out of four of the liver portal lymph node (Figure 2(J2)), two out of four of the mesenteric lymph node (Figure 2(I2)), and two out of four of the Inguinal lymph node (Figure 2(K2)) showed severe to mild hemorrhage. All spleens (Figure 2(L2)) were swollen and/or showed necrosis at the edge zone. Two out of four of the lungs exhibited lobular pneumonia, hemorrhage, or mottled tan consolidation (Figure 2(M2)).

### 3.5. Histopathology Change Caused by PCV3 Infection

**Figure 3:**
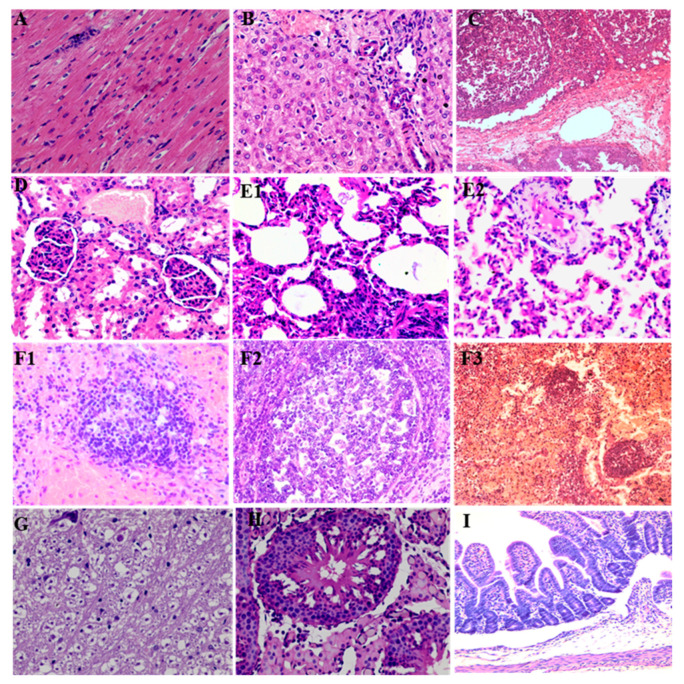
The histopathology change in hearts (**A**), livers (**B**), spleen (**C**), kidney (**D**), lung (**E1**,**E2**), lymph nodes (**F1**–**F3**), brain (**G**), and testis (**H**), small intestine (**I**).

Histopathology examination results showed in the PCV3-inoculated group, the cardiac myocytes were slightly edematous and showed inflammatory cells infiltrated (Figure 3A); the liver showed hepatocyte degeneration, hyperemia, and inflammatory cell infiltration (Figure 3B); the spleen trabecula displayed edematous and widening, and the white pulp of spleen showed bleeding, lymphocyte necrosis, and reduced number of lymphocytes (Figure 3C); the kidney displayed mild tubular epithelial cell necrosis and hyperemia (Figure 3D); the alveoli collapsed, the alveolar wall showed thickening and inflammatory cell infiltration, and some area of the lung showed hyperemia and consolidation (Figure 3(E1,E2)); the lymph nodes (inguinal lymph node, submaxillary lymph node, mesenteric lymph node, hilar lymph node, and liver portal lymph node) all showed bleeding and reduced number of lymphocytes in cortex area (Figure 3(F1–F3)); the brain displayed swelling of nerve cells (Figure 3G); the testis showed spermatogenic epithelium necrosis, interstitial edema and inflammatory cell infiltration (Figure 3H); the small intestine showed submucosal edema (Figure 3I).

### 3.6. The PCV3 in Various Tissues and Organs

As shown in Table 4, PCV3 was also detected by qPCR in all tissue samples evaluated at 42 days p.i. Table 4 summarizes the percentage of PCV3-positive tissues detected in the PCV3-inoculation group at 42 days p.i. The genome copies μL^−1^ detected within different tissues showed that the lymph nodes were the tissues with the highest PCV3 copy numbers; the brain, small intestine and testis were the tissues with no PCV3 infection. The spleen, submaxillary lymph node, and hilar lymph node in all four piglets from the PCV3-inoculation group showed PCV3 positive. However, only two out of four of the piglets’ heart, liver, lung, kidney, tonsil, inguinal lymph node, and mesenteric lymph node showed PCV3 positive. Three out of four of the piglets’ liver portal lymph node showed PCV3 positive.

### 3.7. The Effect of PCV3 Infection on Cytokine Expression Levels in Serum

**Figure 4:**
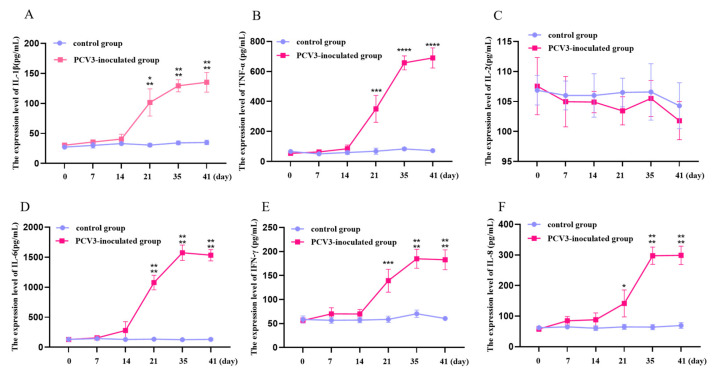
The production of cytokines in serum between the control group and PCV3-inoculated group. **A**: The expression level of IL-1β; **B**: The expression level of TNF-a; **C**: The expression level of IL-2; **D**: The expression level of IL-6; **E**: The expression level of IFN-γ; **F**: The expression level of IL-8. * means *p* < 0.05, ** means *p* < 0.01, *** means *p* < 0.005, **** means *p* < 0.0001 when comparing the PCV3-inoculted group with the control group.

To further confirm that PCV3 infection did cause inflammation response in piglets, we analyzed the cytokines expression levels in serum. As shown in Figure 4, the levels of IL-1β, TNF-α, IL-6, IFN-γ, and IL-8 in PCV3-inoculated groups was increased significantly from 21 day when compared to control group. The levels of IL-1β and TNF-α in PCV3-inoculated groups continued to increase during PCV3 inoculation. The level of IL-6, IFN-γ, and IL-8 in PCV3-inoculated groups reached a peak at 35 days of infection. However, the PCV3 infection did not significantly change the IL-2 expression level in serum.

## 4. Discussion

Since PCV3 was first reported in 2016, lots of studies have proved that PCV3 pathogen has become a common pathogen with a potential threat to the swine populations worldwide [10,20,21,22]. Numerous studies relating to PCV3 have been published recently. The majority of studies, however, have concentrated on the discovery and evolutionary analysis of viral genomes. Studies on PCV3’s pathogenicity and clinical connection remain poorly understood and divisive. The inconsistent origin and caliber of the experimental pigs may be to blame for this. Consequently, in order to reduce the experimental errors caused by the difference in experimental pig breeds, in this study, SPF piglets were chosen for PCV3 infection experiments, which aimed to understand the clinical manifestations and pathogenicity of PCV3 infected piglets under laboratory conditions, and to provide a theoretical basis for the clinical correlation of PCV3 infection in the field.

Even though PCV3 has been extensively reported to be linked to a variety of clinical symptoms, recent laboratory research on PCV3 inoculation in pigs have not revealed any notable clinical signs [23]. In our study, PCV3 was administered to 4 SPF piglets at the age of 4 weeks. The clinical symptoms such as PDNS were not obvious. However, the piglets in the PCV3-inoculation group showed fever, loss of weight, loss of appetite, depression, diarrhea, and lameness. These symptoms are similar to post-weaning multisystemic wasting syndrome (PMWS) which manifests as a clear progression of emaciation or retardation. However, other PMWS symptoms such as pale skin, uncombed coat, dyspnea, and respiratory disturbances characterized by cough were not observed in the PCV3 group [24]. Nevertheless, compared with the above-mentioned PCV3 clinical disease observed under experimental conditions, our experiments are the first time that the inoculated piglets displayed clinical signs using PCV3 isolated strain.

Clinical studies have shown that viremia can persist for a long time with a positive rate of between 6.5 and 25% throughout the production cycle, indicating the possibility of long-term persistent infection [24]. The studies assessed pigs regularly from weaning to slaughter at four different farms. Field cases suggest that the reduced viral load in older pigs [25] may be due to immunity built up after multiple exposures to viruses throughout the reproductive cycle. Similarly, younger boars under 12 months had much greater virus loads than older boars [26]. The lab study results showed that persistent viremia can be detected at at least 28 dpi [27] and in some cases, confirmed after 42 dpi [17]. Moreover, the earliest viremia can be detected at three days post-infection (dpi) [17], the maximum genomic copies of PCV3 reached from 3 dpi to 21 dpi [16,27,28]. Our results showed that the viremia was detected as early as 17 dpi and last at 41 dpi, consistent with the above-mentioned results. Anyway, our observation confirmed that viremia can persist for a long time and these results are consistent with what has been observed in other ssDNA viruses, such as PCV2 [29,30].

Studies have been proven that PCV3 may be transmitted through the nasal and feces. In this study, the PCV3 genome copies of nasal and fecal swabs were detected. The results showed that the piglets-inoculated with PCV3 nasal shedding was detectable from 21 to 35 dpi, the feces shedding occurred intermittent from 21 to 39 dpi. However, Temeeyasen et al. [17] suggest that in experimentally inoculated pigs, intermittent nasal and feces shedding was detectable from 3 to 28 dpi, earlier and longer than our results. This might occur as a result of the varied titers and strains utilized in the two investigations.

To further study the pathogenesis and tissue tropism of PCV3 infection, we first autopsy PCV3 infected pigs. The heart, kidney, brain, testis, and tonsils do not exhibit notable macroscopic lesions. Hyperemic, festering focal, hemorrhage, swelling and necrosis, lobular pneumonia, or mottled tan consolidation were observed in the liver, spleen, lymph nodes, and lung. The viral load in the organs of piglets inoculated with PCV3 was determined by fluorescence quantitative PCR. The pathogen of PCV3 was detected in most of the tissues, including lung, spleen, lymph nodes, tonsil, heart, liver, and kidney, indicating the diverse tissue dependency of PCV3. Among them, the lymph nodes had the highest PCV3 virus genome content, indicating that PCV3 can more effectively replicate its virus genome in the lymph nodes, and also indicating that the lymph nodes are the most prevalent PCV3 virus investigation and detection.

Further details about the histopathological challenges brought on by PCV3 infection were provided. The heart, liver, and lungs were infiltrated by inflammatory cells, consistent with the clinical cases of PCV3 infection in pigs which are marked by multisystemic inflammation [5]. However, when compared with Mora-Díaz et al. [16] results, even though we both observed multisystemic inflammation, the inflammation showed in different tissues and organs. We did not observe interstitial nephritis, periarteritis and arteritis. That may be due to the different species of piglets and different PCV3 strains we used in the experiments. The spleen and lymph node showed lymphocyte necrosis and reduced number of lymphocytes, suggesting that PCV3 infection have damaged the immune system of pigs, leading to immune suppression of pigs. In addition, the alveoli collapsed, consolidation, epithelial cell necrosis, hyperemia, edema, and hemorrhage also observed in all PCV3 positive tissues. The histological abnormalities in the spleen and lung are similar to post-weaning multisystemic wasting syndrome (PMWS) which also showed pulmonary consolidation, enlargement of at least one lymph node. However, we did not observe lymphatic depletion, histiocytic infiltration, inclusion of bodies, and giant cells in lymphatic tissues in PCV3 group [24]. Interestingly, even though we did not detect PCV3 replication in the brain, testis, and small intestine, the above-mentioned histopathological changes including inflammatory cell infiltration and edema were also observed in those tissues. Those results suggest that the tissue lesions caused by PCV3 are not only related to its replication in vivo, but may also be due to the virus-induced inflammatory response.

The pro-inflammatory cytokines have a crucial role in the development and maintenance of inflammation and can lead to damage to multiple organs. Thus, we checked the cytokines expression in pig serum to further prove that the PCV3 infection did cause inflammation response. In the present study, the levels of various pro-inflammatory cytokines IL-6, IFN-γ, and IL-8 in the serum of the PCV3-inoculated piglets peaked at the time of the maximal viremia IL-1β and TNF-α expression persisted arising from 21 days of the experiment. Moreover, the PCV3-mediated clinical symptoms of illness and tissue damage may be caused by the high levels of pro-inflammatory cytokines. Previous studies also proved that PCV3-infection can up-regulate pro-inflammation cytokines in pigs. However, their results showed that levels of cytokines in the serum of the PCV3-inoculated pigs peaked at the time of the maximal viremia [29]. Our results showed some cytokines have the same trend with viremia, some are not. The different results may be caused by the different stains and different species of pigs.

The clinical manifestations of pigs infected with PCV3 in this study were less severe than those of clinical cases and no piglets died during the study, indicating that the pathogenicity of PCV3 may be a little bit weak in the lab condition. Indicating the appearance of clinical symptoms and the severity of the disease in situ, PCV3 may be influenced by multiple factors such as the immune status of the pig population, stress factors, or co-infection with other pig pathogens. In this study, PCV3 infection caused lymphocytopenia in porcine lymph nodes and spleen, so the promoting effect of mixed infection on pathogenicity and morbidity of PCV3 cannot be ruled out. Whether PCV3 can be used as an additional pathogenic factor to promote the production of pathogenic effects needs further study.

## 5. Conclusions

PCV3 has been present in swine populations all over the world for at least the past 20 years, according to a number of retrospective studies [9], but its clinical manifestations did not come to attention until the first description in 2016. In this study, the potential role of PCV3 in subclinical infections was demonstrated, with mild multisystemic inflammation, decreased lymphocytes in immune organs, prolonged viremia detectable for 42 days, and viral shedding in nasal secretions. These findings provide insight into the pathogenesis and inflammation response of PCV3-related diseases. More research is necessary to comprehend and elucidate any necessary cofactors of clinical disease presentation and severity, viral immune avoidance strategies, and the economic impact of PCV3 infection.

## Figures and Tables

**Table 1:** Results of PCV3 detection by qRT-PCR in serum by PCV3 pigs.

The PCV3 Genome Copies of Serum in Two Groups (Copies/μL)																	
Days of post inoculation	0	3	7	10	14	17	21	23	25	27	29	31	33	35	37	39	41
control group (All piglets)	-	-	-	-	-	-	-	-	-	-	-	-	-	-	-	-	-
PCV3-inoculated groups No.1	-	-	-	-	-	10^2.05^	10^2.24^	10^2.48^	10^2.81^	10^3.13^	10^3.55^	10^3.47^	10^3.85^	10^4.17^	10^4.16^	10^4.17^	10^4.12^
PCV3-inoculated groups No.2	-	-	-	-	-	-	10^2.03^	10^2.22^	10^2.42^	10^2.59^	10^3.02^	10^3.25^	10^4.17^	10^4.25^	10^4.08^	10^4.09^	10^4.16^
PCV3-inoculated groups No.3	-	-	-	-	-	-	10^2.26^	10^2.49^	10^2.69^	10^2.83^	10^3.26^	10^3.40^	10^4.09^	10^4.46^	10^4.21^	10^4.02^	10^4.12^
PCV3-inoculated groups No.4	-	-	-	-	-	-	-	10^2.07^	10^2.69^	10^3.11^	10^3.16^	10^3.26^	10^4.14^	10^4.24^	10^4.17^	10^4.11^	10^3.87^
Percentage of positive animals	0%	0%	0%	0%	0%	25%	75%	100%	100%	100%	100%	100%	100%	100%	100%	100%	100%

**Table 2:** Results of PCV3 detection by qRT-PCR in nasal swabs by individual pigs.

The PCV3 Genome Copies of Nasal Swabs in Two Groups (Copies/μL)																	
Days of post inoculation	0	3	7	10	14	17	21	23	25	27	29	31	33	35	37	39	41
control group (All piglets)	-	-	-	-	-	-	-	-	-	-	-	-	-	-	-	-	-
PCV3-inoculated groups No.1	-	-	-	-	-	-	10^1.87^	10^2.08^	10^2.22^	10^2.47^	10^2.68^	10^2.99^	10^2.90^	10^2.35^	-	-	-
PCV3-inoculated groups No.2	-	-	-	-	-	-	10^1.76^	-	-	-	10^2.30^	10^2.73^	10^2.59^	-	-	-	-
PCV3-inoculated groups No.3	-	-	-	-	-	-	-	10^1.85^	10^2.10^	-	10^2.56^	10^2.81^	10^2.86^	10^2.45^	-	-	-
PCV3-inoculated groups No.4	-	-	-	-	-	-	-	-	10^2.08^	10^2.38^	10^2.65^	10^2.93^	-	10^2.39^	-	-	-

**Table 3:** Results of PCV3 detection by qRT-PCR in fecal swabs by individual pigs.

The PCV3 Genome Copies of Fecal Swabs in Two Groups (Copies/μL)																	
Days of post inoculation	0	3	7	10	14	17	21	23	25	27	29	31	33	35	37	39	41
control group (All piglets)	-	-	-	-	-	-	-	-	-	-	-	-	-	-	-	-	-
PCV3-inoculated groups No.1	-	-	-	-	-	-	10^1.96^	10^2.13^	10^2.07^	10^1.86^	-	-	-	10^1.96^	-	10^1.78^	-
PCV3-inoculated groups No.2	-	-	-	-	-	-	-	10^1.88^	-	-	10^1.96^	-	10^1.84^	-	-	-	-
PCV3-inoculated groups No.3	-	-	-	-	-	-	-	-	10^1.90^	10^2.00^	-	10^1.86^	10^1.90^	-	-	-	-
PCV3-inoculated groups No.4	-	-	-	-	-	-	-	10^1.94^	-	-	10^1.80^	10^2.00^	-	-	-	10^1.93^	-

**Table 4:** Summary of PCV3 loads in the various by tissues qRT-PCR at 42 days infection.

42 Days Infection of PCV3				
Tissues	The Genome Copies in PCV3-Inoculated Groups (Copies/μL)	Percentage of Positive Tissues in 4 Piglets of PCV3-Inoculated Groups	Control Group	Percentage of Positive Tissues in 4 Piglets of Control Group
Heart	10^2.35^ ± 64.19	50%	-	0%
Liver	10^3.28^ ± 145.49	50%	-	0%
Spleen	10^3.88^ ± 609.79	100%	-	0%
Lung	10^3.38^ ± 93.64	50%	-	0%
Kidney	10^2.35^ ± 140.27	50%	-	0%
Tonsil	10^3.47^ ± 1561.79	50%	-	0%
Inguinal lymph node	10^4.12^ ± 898.445	50%	-	0%
Submaxillary lymph node	10^4.48^ ± 7072.02	100%	-	0%
Mesenteric lymph node	10^4.35^ ± 8088.98	50%	-	0%
Hilar lymph node	10^4.18^ ± 7215.42	100%	-	0%
Liver portal lymph node	10^4.19^ ± 4424.75	75%	-	0%
Brain	-	0%	-	0%
small intestine	-	0%	-	0%
Testis	-	0%	-	0%

## Data Availability

The data that support the findings of this study are available from the corresponding author.
